# Biochemical responses, feeding and survival in the solitary bee *Osmia bicornis* following exposure to an insecticide and a fungicide alone and in combination

**DOI:** 10.1007/s11356-022-24061-x

**Published:** 2022-11-16

**Authors:** Cátia Ariana Henriques Martins, Ilaria Caliani, Antonella D’Agostino, Agata Di Noi, Silvia Casini, Martina Parrilli, Celeste Azpiazu, Jordi Bosch, Fabio Sgolastra

**Affiliations:** 1 Department of Agricultural and Food Sciences, Alma Mater Studiorum Università Di Bologna, Viale Fanin 42, 40127 Bologna, Italy; 2 Department of Physical, Earth and Environmental Sciences, University of Siena, Via Mattioli 4, 53100 Siena, Italy; 3 Department of Management and Quantitative Studies, University of Naples Parthenope, Naples, Italy; 4 Department of Life Sciences, University of Siena, Via Mattioli, 4, 53100 Siena, Italy; 5 Institute of Evolutionary Biology (CSIC-Universitat Pompeu Fabra), Passeig Marítim de La Barceloneta 37, 08003 Barcelona, Spain; 6 Universidad Politécnica de Madrid, 28040 Madrid, Spain; 7 CREAF, Universitat Autònoma de Barcelona, 08193 Barcelona, Bellaterra Spain

**Keywords:** Biomarkers, IBRv2 index, Imidacloprid, Pesticides, Sub-lethal effects, Tebuconazole

## Abstract

In agricultural ecosystems, bees are exposed to combinations of pesticides that may have been applied at different times. For example, bees visiting a flowering crop may be chronically exposed to low concentrations of systemic insecticides applied before bloom and then to a pulse of fungicide, considered safe for bees, applied during bloom. In this study, we simulate this scenario under laboratory conditions with females of the solitary bee, *Osmia bicornis* L. We studied the effects of chronic exposure to the neonicotinoid insecticide, Confidor® (imidacloprid) at a realistic concentration, and of a pulse (1 day) exposure of the fungicide Folicur® SE (tebuconazole) at field application rate. Syrup consumption, survival, and four biomarkers: acetylcholinesterase (AChE), carboxylesterase (CaE), glutathione S-transferase (GST), and alkaline phosphatase (ALP) were evaluated at two different time points. An integrated biological response (IBRv2) index was elaborated with the biomarker results. The fungicide pulse had no impact on survival but temporarily reduced syrup consumption and increased the IBRv2 index, indicating potential molecular alterations. The neonicotinoid significantly reduced syrup consumption, survival, and the neurological activity of the enzymes. The co-exposure neonicotinoid-fungicide did not increase toxicity at the tested concentrations. AChE proved to be an efficient biomarker for the detection of early effects for both the insecticide and the fungicide. Our results highlight the importance of assessing individual and sub-individual endpoints to better understand pesticide effects on bees.

**Supplementary Information:**

The online version contains supplementary material available at 10.1007/s11356-022-24061-x.

## Introduction

Pesticide use associated with agricultural intensification is considered one of the main drivers of pollinator declines (Goulson et al. 2015). Although most studies focus on single products or active ingredients, pollinators are usually exposed to combinations of products (Woodcock et al. 2016; Grab et al. 2019). Multi-pesticide exposure may occur due to the application of tank mixtures, but also when different products are applied sequentially. For example, residues of systemic insecticides from treatments performed before bloom (e.g., as seed coating) may appear in the flowers and get mixed with fungicides applied during bloom. Due to their low toxicity for bees, many entomophilous crops are sprayed with fungicides at least once during bloom (Xavier et al. 2020; Almasri et al. 2021). Under this scenario, pollinators experience chronic exposure to residual concentrations of systemic insecticides and acute exposure to high concentrations of fungicides. The levels of insecticides applied before bloom appearing in the pollen and nectar of crop flowers are typically low (Zioga et al. 2020). Some studies have reported that such concentrations pose no lethal risk to bees (Maus et al. 2003; Faucon et al. 2005; Nguyen et al. 2009), but may cause sub-lethal effects. On the other hand, fungicides are not supposed to directly harm insects, but sub-lethal effects, including genotoxicity (Caliani et al. 2021a) and alterations of the feeding behavior (Zhu et al. 2017a), have been found in *Apis mellifera*. In addition, several studies have demonstrated that some insecticide-fungicide combinations induce synergistic toxicity effects in bees (Pilling et al. 1995; Thompson and Wilkins 2003; Johnson et al. 2013; Thompson et al. 2014; Mengoni Goñalons and Farina 2018; Wang et al. 2020a, b a,b). In particular, sterol biosynthesis inhibiting (SBI) fungicides have been shown to interact with neonicotinoids (Iwasa et al. 2004; Biddinger et al. 2013; Sgolastra et al. 2017; Raimets et al. 2018; Iverson et al. 2019). This interaction occurs because SBI fungicides modify the metabolic detoxification processes in bees by inhibiting cytochrome P450-monooxygenase (Berenbaum and Johnson 2015; Carnesecchi et al. 2019).

Sub-lethal effects are not easy to detect over the course of customary toxicological tests in the laboratory. Syrup consumption is an easy-to-measure fitness endpoint that may provide insights on pesticide-induced changes at the individual level. At the sub-individual level, the detoxification energy costs related with enzymatic activity may have repercussions on fitness-related traits (Castañeda et al. 2009). For this reason, biomarkers, which can provide signals of early stage alterations at lower biological levels, represent an important tool to evaluate sub-lethal effects (Caliani et al. 2021a). Different biochemical and cellular biomarkers have been developed and applied in honey bees to assess ecotoxicological health status and the sub-lethal effects of different pollutant compounds such as pesticides, heavy metals, and PAHs (Badiou-Bénéteau et al. 2012; Carvalho et al. 2013; Zhu et al. 2017b; Han et al. 2019; Caliani et al. 2021b). Acetylcholinesterase (AChE) and carboxylesterases (CaE) have been widely used as biomarkers to assess the effects of different insecticide classes, such as organophosphates and carbamates, since they mechanistically interact with the nervous tissues of organisms (Sanchez-Hernandez 2011). Other important biomarkers, such as glutathione S-transferase (GST) and alkaline phosphatase (ALP), are involved in the biotransformation and detoxification of pollutants, and were first appointed as good candidates to monitor the defenses of the honey bee by a neonicotinoid insecticide (Badiou-Bénéteau et al. 2012). In the last years, the search for biomarkers indicative of sub-lethal effects to various organisms has become a priority in ecotoxicological research (Tlili and Mouneyrac, 2021; López-Uribe et al. 2020); however, most studies on bees have only targeted the western honey bee, *A. mellifera*, and studies on solitary bees are mostly lacking (Mokkapati et al. 2022). A research effort on this topic is fundamental because solitary bees are more sensitive than honey bees to certain pesticides (Arena and Sgolastra 2014; Sgolastra et al. 2017; Azpiazu et al. 2021) and have different routes and levels of exposure (Sgolastra et al. 2019). In fact, the European Food Safety Authority pointed out the necessity to include *Osmia* spp. as representative species of solitary bees in pesticide risk assessment (EFSA 2013). *Osmia bicorni*s is common European solitary bee that is managed for crop pollination in some areas (Sedivy and Dorn 2014), and therefore is often exposed to pesticides.

In this study, we conducted a laboratory experiment in which we combined a chronical exposure to a field-realistic concentration of an insecticide (Confidor®, imidacloprid) with a single exposure to a fungicide (Folicur® SE, tebuconazole) in females of the solitary bee *O. bicornis*. Despite the ban on the use of neonicotinoids (imidacloprid, thiamethoxam, and clothianidin) in the European Union, their presence in the environment is still reported due to the high persistence of neonicotinoids (Botías et al. 2016; Wintermantel et al. 2020), and therefore they may still pose a threat to pollinators. In addition, neonicotinoids are still widely used in non-EU countries (Goulson 2020). We thus simulated a scenario in which bees foraging on flowers with residual concentrations of a systemic insecticide are exposed to a high fungicide dose applied during bloom. Our study has important implications for pesticide risk assessment: first, current risk assessment schemes are mostly based on single compounds (Rortais et al. 2017), even in the face of increasing evidence that pollinators are exposed to mixtures of pesticides (Sgolastra et al. 2020); second, current risk assessment schemes mostly overlook sub-lethal effects such as behavioral and physiological responses that may affect bee health even when no effects on survival are detected (Cresswell 2011; Azpiazu et al. 2019; Sandrock et al. 2014); third, pesticide risk assessment has traditionally relied on a single species, the western honey bee, although pesticide effects may be species-dependent (Schmolke et al. 2021), and extrapolation from honey bees to wild bees may not adequately reflect realistic scenarios due to colony resilience in honey bees (Rundlöf et al. 2015).

In this study, we measured syrup consumption and survival at the individual level, as well as a set of biomarkers covering various biological responses, including neurotoxicity (AChE and CaEs) and metabolic activity (GST and ALP). We also propose the development of an integrated biological response (IBRv2) index (Sanchez et al. 2013) providing a measure of the overall response of the target organism to the exposure of pesticides in *O. bicornis*. This index is based on the biomarker deviation from the reference site, allowing the identification of how each selected biomarker contributes to the final toxicological status (Arrighetti et al. 2019). To our knowledge, IBRv2 indexes have not been developed for insect pollinators except for honey bees (Caliani et al. 2021a, b a,b), but they are widely used to investigate the effects of different contaminants on other groups of organisms. Our goal was to assess whether the chronic exposure to the insecticide, the fungicide pulse, and the insecticide-fungicide combination elicited some biomarker responses that could be related to syrup consumption and survival.

## Material and methods

### Pesticides

We used commercially available formulates, Confidor® (imidacloprid 20% w/v) and Folicur® SE (tebuconazole 4.35% w/v), rather than active ingredients. The two pesticides were chosen because they are extensively used for pest and disease control in bee-pollinated crops such as fruits, nuts, and vegetables. Many studies have documented co-occurrence of the two active ingredients in nectar and pollen samples (Chauzat et al. 2006, 2009, 2011; Mullin et al. 2010; Pohorecka et al. 2012; David et al. 2015, 2016; Lentola et al. 2017; Ostiguy et al. 2019).

Stock solutions of each pesticide were prepared by dissolving the products in distilled water at nominal concentrations of 50 µg L^−1^ of Confidor® and 1850 mg L^−1^ of Folicur® SE. The stock solutions were then diluted in a feeding solution (sugar and distilled water at 47.5% w/v; henceforth syrup) to achieve the desired concentrations of 5 µg L^−1^ and 185 mg L^−1^ of imidacloprid and tebuconazole, respectively. The final concentration of the syrup given to bees was 38% w/v (Azpiazu et al. 2019). The concentration of imidacloprid was within the range of residues found in nectar collected from flowers of different crops, either grown from imidacloprid-coated seed or treated via soil or spray applications (citrus: 0.8–6.82 ng mL^−1^ [Byrne et al. 2014]; apples: 2–70 ppb [Heller et al. 2020]; cucurbits: 3.8–7.3 ng g^−1^ and 6.7–16 ng g^−1^ [Dively and Kamel 2012], 5–14 ppb [Stoner and Eitzer 2012]; sunflower: 0.0019 (± 0.001) mg kg^−1^ [Schmuck et al. 2001]; ornamental plants: < 1.2–5.7 ng g^−1^ [Lentola et al. 2017]). For tebuconazole, we worked with the potential concentration immediately after spray application, calculated as the maximum field application rate of its commercial formulation (6.45 L ha^−1^) in orchards.

### Osmia bicornis and test conditions

Bees were supplied by Pollinature Srl. Cocoons were shipped to the Department of Agricultural and Food Sciences, University of Bologna, Italy, and kept at wintering temperatures of 3–4 °C and 55 ± 10% relative humidity. In May 2021, large cocoons, expected to be females, were incubated at 22–23 °C until emergence. We worked with newly emerged females (< 24 h old). Over a period of 5 days, emerging females were distributed randomly and equally among the four exposure treatments (see below). Upon emergence, females were transferred to a Plexiglas flight cage (50 × 50 × 50 cm) for meconium deposition and 24 h starvation. Two hundred and forty bees (60 bees per treatment) were then transferred to individual cages (transparent plastic cups; volume: 150 cc), with perforated lids to allow air circulation. Each cup was provided with a syrup feeder consisting of a 1-mL-calibrated syringe (BEROJECT® III, accuracy: 0.02 mL) inserted laterally and slightly inclined. A petal of *Euryops* (Asteracea) was attached to the tip of the syringe to enhance prompt location of the feeder by the bee (Sgolastra et al. 2018; Azpiazu et al. 2019). From emergence until death, bees were maintained at 21–24 °C and 50–55% relative humidity under natural light, avoiding direct sunlight to reduce pesticide degradation.

### Exposure conditions

After 24 h of starvation, bees were divided into 4 groups: control (CTRL), insecticide Confidor® (INS), fungicide Folicur® SE (FUNG), and the two pesticides (MIX). Bees of the CTRL treatment were fed regular syrup throughout the experiment. Bees of the FUNG treatment were also fed regular syrup throughout the experiment except on day 3 when they were offered syrup with fungicide. Bees of the INS treatment were fed syrup with insecticide throughout the experiment. Bees of the MIX treatment were also fed syrup with insecticide throughout the experiment, except on day 3 when they were fed syrup with insecticide and fungicide. In the treatment groups FUNG and MIX, the solution with fungicide was only offered for a period of 24 h to simulate a pulse exposure. This exposure scenario represents a compromise between a worst-case scenario, which does not account for fungicide degradation during the 24 h, and a best-case scenario in which the fungicide is completely degraded in one day. Tebuconazole is known to be a stable compound under hydrolytic and photolytic conditions (Lewis et al. 2006; EFSA 2014). In all cases, bees were fed ad libitum throughout the experiment. To avoid fungal proliferation, feeding solutions were freshly prepared every 3 days.

### Syrup consumption and survival

Syrup consumption and survival were monitored daily until all bees died. To account for potential evaporation, syrup levels were measured in eight cages without bees. After dead, the head width of each bee was measured under a stereomicroscope with a micrometer as a proxy of body size (Bosch and Vicens 2002).

### Collection of tissue samples

Ten bees per treatment were collected for biomarker assessment at two different time points: T1 (on the fourth day of exposure, that is 24 h after the fungicide pulse) and T2 (on the sixth day of exposure, that is 72 h after the fungicide pulse). Bees were anesthetized in ice (4 °C) for 30 min and then the midgut and the head were removed and immediately frozen at − 80 °C.

### Biomarker analysis

For each specimen, the head and midgut were processed separately to obtain the extracts on which to perform the enzymatic tests. Nervous tissue extracts from the head were used to evaluate AChE and CaE, and midgut extracts were used to measure GST and ALP activities. Tissues were weighted, and extraction medium was added proportionally to the weight of the tissue at a ratio of 10% (w/v). The buffer contained 40 mM Na phosphate buffer (pH 7.4), a mixture of protease inhibitors enzymes and 1% Triton X-100. The samples were homogenized by a tissuelyser (Qiagen) at 20 F for three periods of 30 s at 30-s intervals. The homogenates were centrifuged at 4 °C for 20 min at 13,000 g and 15,000 g for head and gut samples, respectively. The resulting supernatants were frozen at − 80 °C and used for the analyses.

#### AChE

The AChE activity was assayed in the head extracts according to Ellman et al. (1961) with modification from Caliani et al. (2021b). The reaction mixture was prepared in a 3-mL cuvette and contained 0.1 M sodium phosphate buffer (pH 7.4), 10 mM DTNB, 41.5 mM acetylthiocholine, and 5 μL head extract. The activity was monitored continuously with a spectrophotometer (Agilent CARY UV60) for 5 min at 410 nm (25 °C) and expressed in μmol^−1^ g tissue^−1^ min.

#### CaE

The CaE activity was measured in the head extracts and quantified at 538 nm according to Caliani et al. (2021a). A mixture containing 100 mM sodium phosphate buffer (pH 7.4) and a 0.1-mL head extract was prepared and incubated at 25 °C for 5 min. The reaction was started by adding 0.4 mM α-NA as a substrate. After 3 min, the reaction was stopped adding 1.5% SDS and 0.4 mg/L Fast Garnet GBC. The products of the reaction were quantified spectrophotometrically (Agilent CARY UV60) at 538 nm (25 °C) and the enzyme activity was expressed as nmol α-NA min^−1^ mg^−1^ protein (*ε* = 23.59 × 103 mM^−1^ cm^−1^).

#### GST

The GST activity was measured in the midgut samples following the method of Habig et al. (1974), modified. The reaction mixture consisted of 0.1 M sodium phosphate buffer (pH 7.4), 8 mM GSH (reduced glutathione), 8 mM CDNB, and 30 μL extract. The conjugation of GSH with 1-chloro-2,4-dinitrobenzene (CDNB) was recorded spectrophotometrically (Agilent CARY UV60) at 340 nm (25 °C) and expressed as nmol CDNB conjugate formed min^−1^ mg^−1^ protein (*ε* = 9.6 × 103 mM^−1^ m^−1^).

#### ALP

The ALP activity was assayed in the midgut samples following the formation of p-nitrophenol, a product of the hydrolysis of the substrate (PNPP) due to the enzyme’s activity, according to Bounias et al. (1996), modified. The reaction mixture consisted of 100 mM Tris–HCl buffer (pH 8.5), 100 mM MgCl2, 100 mM p-NPP as the substrate and a 25-μL gut extract. The reaction was monitored continuously for 5 min at 405 nm (25 °C) at the spectrophotometer (Agilent CARY UV60), and the activity was expressed as nmol p-nNPP min^−1^ mg^−1^ protein (*ε* = 18.81 × 103 mM^−1^ cm^−1^ cm^−1^).

#### Protein concentrations

Protein concentrations were measured according to the method of Bradford (1976) by BioRad Protein Assay (BioRad), using bovine serum albumin (BSA) as standard.

### Statistical analysis

Statistical analysis was carried out with STATA (StataCorp 2015) and data visualization with R software (Team R Core 2013). Only bees that consumed at least 10 µL on the first 2 days of exposure were included in the analyses. Individuals collected for biomarker analysis were not included in the syrup consumption and survival analyses.

To avoid confounding effects of reduced syrup consumption due to aging, we used daily syrup consumption data only up to the median survival date of each treatment. Differences in daily consumption among treatments were analyzed at three different times (the first 2 days of exposure or “pre-pulse,” the day of fungicide exposure or “pulse,” and between the day after fungicide exposure until the median survival date “post-pulse”). We used the Kruskall-Wallis (KW) non-parametric test to detect differences among treatments in daily syrup consumption at each time. Dunn’s test (with Benjamini–Hochberg correction) was performed for pairwise multiple-comparison. A Mann–Whitney *U* test for paired samples was used to test for differences among pre-pulse and post-pulse periods and treatments. Bees that died before the post-pulse period were excluded from this analysis.

Survival functions $S(t)$ were estimated using a Kaplan-Maier estimator with no censoring. Accordingly, $S(t)$ was estimated as $1-{F}_{n}(t)$, where ${F}_{n}(t)$ is the empirical cumulative distribution function.

The comparison of survival rates between CTRL and the other treatments was performed using the Fleming–Harrington test, belonging to the weighted log-rank test ${G}^{\rho ,\lambda }$ class (Fleming and Harrington 2011). We used ${G}^{\mathrm{1,1}}$ to detect differences between treatments especially in the intermediate section of the survival curves.

We performed a Kernel regression (KR) to detect a possible relationship between body size (measured as head width) and syrup consumption, and Cox regression model to detect the potential effect of body size on survival time.

Biomarker data were first analyzed by comparing the median of the two collection times (T1 and T2) for each biomarker and treatment. KW non-parametric test and Dunn’s test were conducted. Spearman’s rank correlation coefficient was used to explore the relationship between pairs of biomarkers. Lastly, integrated biological response (IBRv2) index (Sanchez et al. 2013) was used to quantify in a single value the overall degree of response to each treatment, in which higher IBRv2 values represent a higher stress level. Results are reported with a significance level of 5%.

## Results

### Syrup consumption

**Fig. 1:**
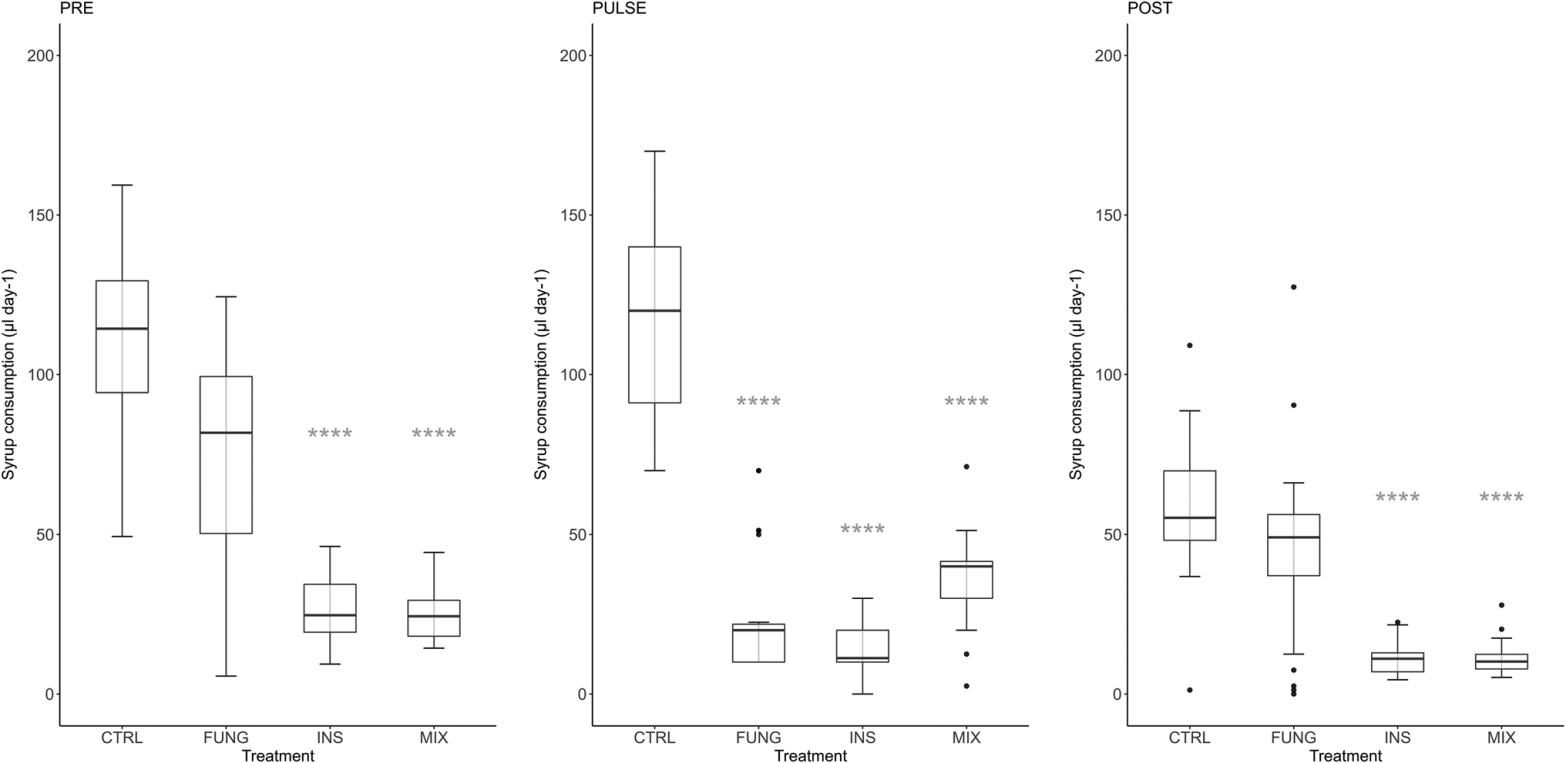
Daily syrup consumption (µl day^−1^) up to the date of 50% mortality within each treatment. CTRL, control (*n* = 25); FUNG, tebuconazole (*n* = 26); INS, imidacloprid (*n* = 26); MIX, tebuconazole + imidacloprid (*n* = 24)l PRE, first 2 days of exposure; PULSE, day 3; POST, after the 3.^rd^ day up to the date of 50% mortality within each treatment. Boxplots with asterisks are significantly different from the control (Dunn’s pairwise comparison, ****p < 0.0001)

Significant differences in syrup consumption were found between treatments with and without the insecticide (Dunn’s test; *p* ≤ 0.0001; Fig. 1 and Table A.1); overall, bees from INS and MIX consumed approximately 74% less syrup than bees of the CTRL and FUNG treatments. The fungicide pulse (FUNG) caused a decrease in feeding rate, which returned to control levels over the post-pulse period. Overall, syrup consumption significantly (*p* < 0.0001) decreased from the pre-pulse to the post-pulse assessments in all treatments (see Table A.2 for Mann–Whitney *U* test results). Kernel regression analysis indicates that body size had no effect on daily syrup consumption (Table A.3).

### Survival analysis

**Fig. 2:**
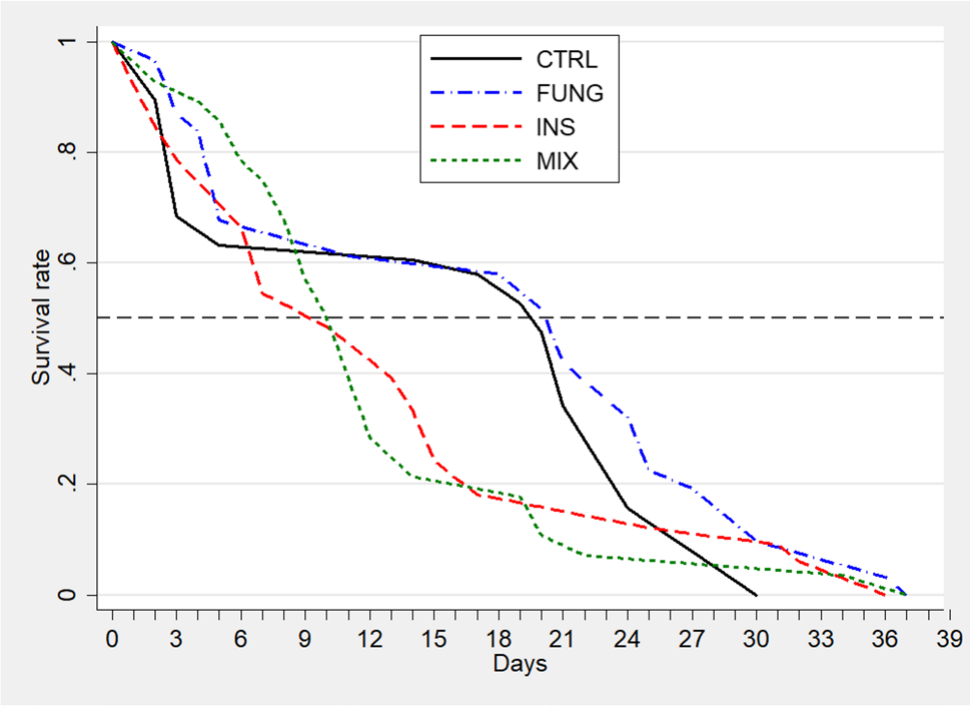
Survival curves of *Osmia bicornis* females orally exposed to various pesticide treatments. CTRL, control (*n* = 38); FUNG, tebuconazole (*n* = 31); INS, imidacloprid (*n* = 33); MIX, tebuconazole + imidacloprid (*n* = 27). The dashed line indicates 50% survival rate

Exposure to INS and MIX had an effect on survival of *O. bicornis* females. Survival significantly differed among treatments (*p* < 0.01) in the intermediate part of the distribution curves (Fig. 2 and see Table A.4 for results of Fleming–Harrington tests). Median survival time dropped from approximately 21 days for CTRL and FUNG bees to 11 days for INS and MIX bees. Body size had no effect on survival (see Table A.5 for Cox model results).

### Biomarkers

**Fig. 3:**
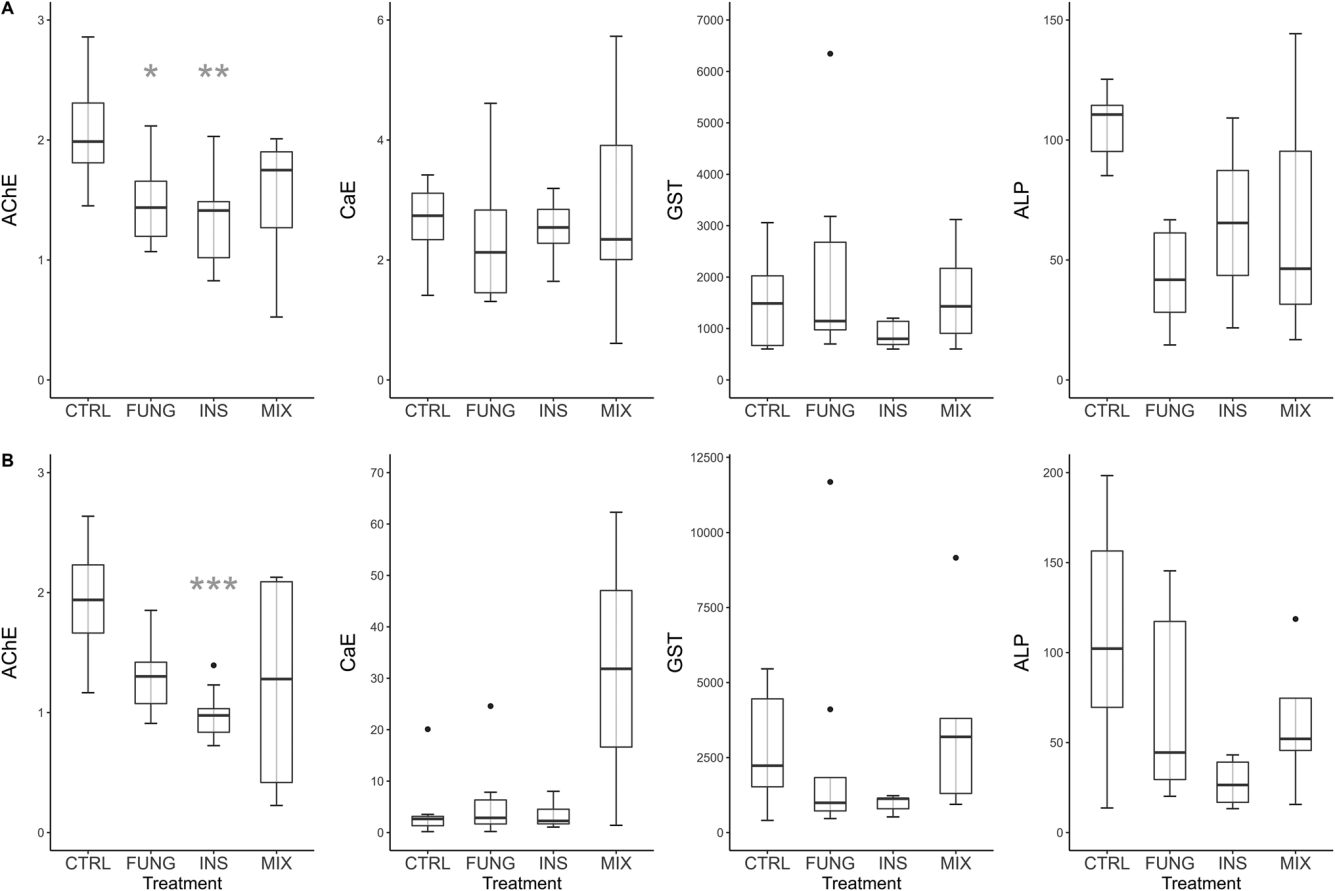
Activity of biomarkers AChE (μmol^−1^ g tissue^−1^ min), CaE (nmol min^−1^ mg^−1^ protein), GST (nmol min^−1^ mg^−1^ protein) and ALP (nmol min^−1^ mg.^−1^ protein) in *Osmia bicornis* females orally exposed to various pesticide treatments. CTRL, control; FUNG, tebuconazole; INS, imidacloprid; MIX, tebuconazole + imidacloprid. Measurements were taken at T1 (day 4 of exposure; **A** and T2 (day 6 of exposure; **B** boxplots with asterisks are significantly different from the control (Dunn’s pairwise comparison, *p < 0.05; **p < 0.01, ***p < 0.001)

Biomarkers of neurotoxicity (AChE and CaE) and metabolic activity (ALP and GST) were assessed on days 4 (T1) and 6 (T2), that is 24 h and 72 h after the fungicide pulse, respectively. The results of the four biomarkers at T1 and T2 are shown in Fig. 3. The results of the descriptive statistics, the Kruskal–Wallis tests for the assessment of statistically significant differences among groups for each biomarker and syrup consumption at T1 and T2, and the Dunn’s pairwise comparison with the control group are summarized in the supplementary material (Tables A.6, A.7 and A.8). AChE activity was significantly inhibited in the INS treatment at both times compared to the control (Dunn’s test; T1, *p* < 0.01; T2, *p* < 0.001); AChE was also significantly inhibited in FUNG treatment at T1 (Dunn’s test; *p* < 0.05). No significant differences were observed for CaE, GST, and ALP activity, in none of the assessment times. Overall, we found a significant positive correlation between ALP and GST at T1 (*p* < 0.05; *ρ* = 0.666) and at T2 (*p* < 0.001; *ρ* = 0.806) and a positive and significant correlation between syrup consumption and AChE activity at T1 (*p* < 0.05; *ρ* = 0.4072) and T2 (*p* < 0.01; *ρ* = 0.4710).

### IBRv2

**Fig. 4:**
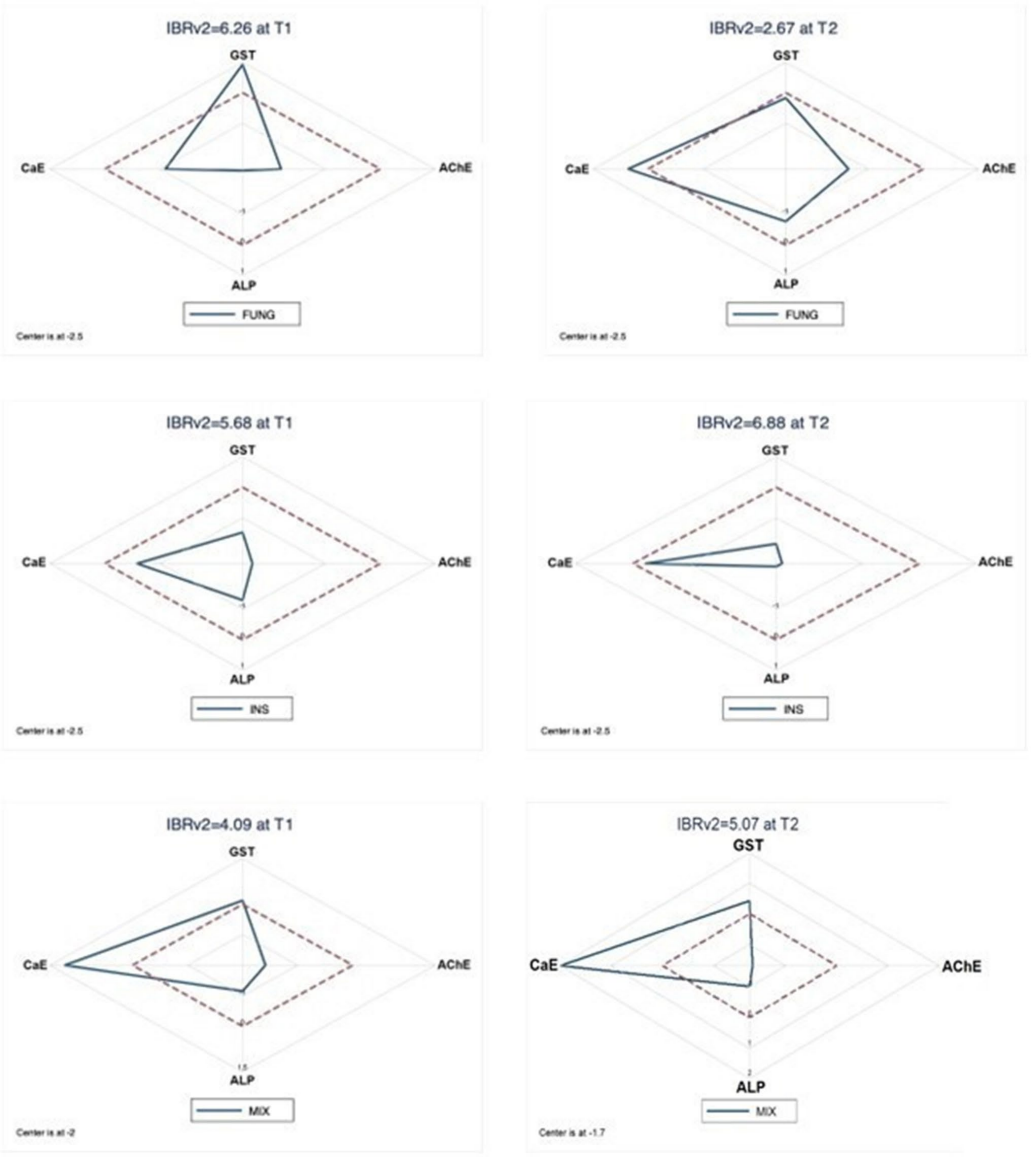
Star plots of the integrated biological response (IBRv2) in *Osmia bicornis* females orally exposed to three pesticide treatments. FUNG, tebuconazole; INS, imidacloprid; MIX, tebuconazole + imidacloprid. Measurements were taken at T1 (day 4 of exposure) and T2 (day 6 of exposure). The dashed line indicates the control values

The results of the integrated biological response (IBRv2) for each treatment are shown in Fig. 4. In the FUNG treatment, the IBRv2 value declined from T1 (6.26) to T2 (2.67). The most discriminant factor for this treatment shifted from GST at T1 to CaE at T2. Bees exposed to INS and MIX showed increasing IBRv2 values from T1 to T2, with CaE as the predominant factor in all the star plots. The MIX treatment showed the lowest IBRv2 at T1 (4.09).

## Discussion

In this study, we tested the effects of oral co-exposure to a neonicotinoid, imidacloprid, and a SBI fungicide, tebuconazole, on adult *O. bicornis* females. Our first objective was to establish whether exposure to a fungicide pulse could enhance the toxicity of low-level chronic exposure to the neonicotinoid. Our second objective was to identify neurotoxicity and metabolic activity biomarkers that could act as early warning signals of sub-lethal effects.

Our results clearly indicate a feeding reduction due to the continued feeding of imidacloprid, which agrees with the findings of other studies (Zhu et al. 2017a; Azpiazu et al. 2019). The fungicide pulse at the maximum field application concentration caused a temporary decrease in feeding rate but did not affect post-pulse syrup consumption. In addition, the fungicide pulse did not impact the feeding of bees of the MIX treatment. A study in which bumble bees were exposed to the combination imidacloprid-imazalil also failed to find synergistic effects on feeding rate (Raimets et al. 2018). The observed effect of imidacloprid on syrup consumption may be related to the reduction of bee mobility, leading bees to ingest less syrup (Medrzycki et al. 2003; Wu et al. 2017).

In addition, the median survival time of bees exposed to imidacloprid at 5 µg L^−1^ (alone and in combination with the fungicide) was significantly shorter (e.g., 10 days) than in bees from CRTL and FUNG treatments. In our study, bees were chronically exposed to a constant concentration of imidacloprid throughout their lifespan, an approach that does not account for pesticide degradation over time or “dilution effect” due to visitation to uncontaminated flowers. However, levels of imidacloprid higher than the concentration tested in our study have been found in the flowers of crop and non-crop plants potentially extending the period of pesticide exposure beyond the blooming of the target crop (Botías et al. 2016; Wintermantel et al. 2020). Interestingly, the toxicity of imidacloprid in our study was higher than in a previous study that used a threefold higher concentration of the same commercial product, Confidor®, on *O. bicornis* females (Azpiazu et al. 2019). Median mortality time in the control bees of the two studies was similar (19 and 20 days, respectively), but the median mortality time of the group treated with imidacloprid was 10 days in our study compared to 16 days in Azpiazu’s study (Azpiazu et al. 2019). These differences could be explained by the different diet offered to the bees. Our bees were provided with syrup only whereas those of Azpiazu et al. (2019) also had access to pollen. Several studies have shown that pollen feeding positively affects health and longevity in honey bees (Pasquale et al. 2013; Huang 2012) and may mitigate the negative impact of pesticides (Castle et al. 2022).

In our study, the fungicide did not affect bee survival, even when combined with the insecticide. Some oral acute exposure studies have found a synergistic effect of the fungicide propiconazole on the toxicity of the neonicotinoid clothianidin (Sgolastra et al. 2018, 2017). In contrast, the tebuconazole pulse did not reduce the survival of *O. bicornis* females chronically exposed to imidacloprid in our study. These results are in line with other studies in which honey bees chronically exposed to imidacloprid-tetraconazole (Zhu et al. 2017b) and imidacloprid-difenoconazole (Pal et al. 2022) mixtures did not yield synergistic effects; similarly, no interactions between imidacloprid (15 µg L^−1^) and myclobutanil were found following chronic oral exposure in O. bicornis (Azpiazu et al. 2019).

We assessed four selected biomarkers (AChE, CaE, GST, and ALP) to determine the impact of the two pesticides at the neurological and metabolic levels. AChE, an important enzyme responsible for the hydrolyses of acetylcholine at the cholinergic synapses (Badiou-Bénéteau et al. 2012), allows the control and modulation of neural transmission (Badiou et al. 2008). In our work, AChE was significantly inhibited by the tebuconazole (27% reduction, at T1) and by the imidacloprid (29% reduction, at T1 and 49% at T2), indicating a clear neurotoxic effect of the two pesticides and confirming AChE as an excellent biomarker for the assessment of sub-lethal effects in *O. bicornis*.

The levels of inhibition can be considered relevant in altering the proper functioning of the nervous system. AChE inhibition has been associated with exposure to some classes of insecticides, such as carbamates and organophosphates (Fulton and Key 2001; Rabea et al. 2010). To date, AChE activity is also used for studying the neonicotinoids and their metabolites neurotoxic effects (Boily et al. 2013; Shao et al. 2013; Samson-Robert et al. 2015; Gyori et al. 2017). As observed by Badawy et al. (2015), neonicotinoids such as dinotefuran (nitro-substituted compound) and acetamiprid (cyano-substituted), enhance the inhibition of AChE activity in honey bees after exposure to different field relevant doses, even though AChE is not the target site of neonicotinoids. In our study, we also observed a neurotoxic effect positively correlated to alterations in the feeding behavior of *O. bicornis*. Caliani et al. (2021b) found a neurotoxic effect of Amistar® Xtra (a.i., azoxystrobin), but no data were produced regarding syrup consumption. The inhibitory effect on the AChE activity by tebuconazole, related with immobility, has been also reported in aquatic organisms (Altenhofen et al. 2017; Lebrun et al. 2021). The use of fungicides may also be associated with sub-lethal effects disrupting the bee’s overall fitness and behavior (Artz and Pitts-Singer, 2015; Fisher et al. 2021). As for the MIX group, in our study, non-statistically significant alterations in this enzyme activity were observed. We can hypothesize the absence of a synergic effect of the two pesticides because we did not observe the highest inhibition in the MIX group. Yet, we cannot exclude an antagonistic or a predominant effect of one compound over the other.

CaE are phase-I detoxifying enzymes that mainly hydrolyse non-polar carboxyl esters (Badiou-Bénéteau et al. 2012; Stone et al. 2002; Barata et al. 2005). Besides, they also play a role in the defense mechanism, protecting AChE from the inactivation caused by organophosphates and carbamates. Several studies have also shown differential expression of CaEs after exposure to pesticides (Badiou-Bénéteau et al. 2012; Zhu et al. 2017a,b). In our study, CaE was not modulated by the fungicide or the insecticide. This result, together with the AChE inhibition, leads us to hypothesize that the AChE was the most affected enzyme.

The main role of the phase-II metabolizing GST isoenzymes is to catalyze the reaction with reduced glutathione (GSH) and conjugate xenobiotic compounds, facilitating their detoxification (Shi et al. 2012). The tendency for the decreased of GST activity, in particular in INS treatment at T1 and T2, could be indicative of an organism’s attempt to respond to an oxidative stress condition. This result could be expected, since imidacloprid is known to induce metabolic disruptions and oxidative stress in honey bees and other animals (Nicodemo et al. 2014; Powner et al. 2016).

ALP is included in the final process of digestion and in the mechanism of active membrane transport (Cheung and Low 1975; Srivastava and Saxena 1967). Although ALP is not involved in detoxification processes, its activity can be modulated in reaction to chemical stress. In our study, the ALP activity was not statistically inhibited by any treatment or time, although we observed an overall decrease in its activity. Other studies showed a modulation of ALP in honey bees exposed to insecticides, such as fipronil, spinosad, imidacloprid, or following infection by *Nosema* (Dussaubat et al. 2012; Carvalho et al. 2013; Kairo et al. 2017; Paleolog et al. 2020). An inhibition was also found by Caliani et al.( 2021a), after honey bee exposure to fungicides and heavy metals. A previous study (Almasri et al. 2020) on honey bees did not find ALP modulation after the administration of combinations of imidacloprid, glyphosate and difenoconazole. We also found a positive correlation between GST and ALP at both times. The positive correlation between these two enzymes could indicate that both are affected by imidacloprid and tebuconazole.

We used the IBRv2 index to integrate the responses of the selected biomarkers (AChE, CaE, GST, and ALP). This approach facilitates the visualization of the spatial arrangement of different enzymatic responses and the possible effects of different contaminant compounds. At T1, the FUNG treatment showed the highest IBRv2 value, followed by the INS treatment and finally the MIX treatment. This result indicates that the fungicide alone induced a high oxidative stress, particularly expressed by GST activity, although no difference was found between treatments. Previous studies also found increased GST response after fungicide exposure (Johansen et al. 2007; Han et al. 2014). Since the fungicide was administered as a pulse, we expected an improvement of the organisms’ health status at T2 that was confirmed by the IBRv2 lowest value. This result suggests that the bees are able to biotransform and detoxify when they are not chronically exposed to the fungicide. On the other hand, the IBRv2 value increased from T1 to T2 in the INS treatment, as expected given the continuous exposure to the pesticide. This treatment group is also the one that shows the highest IBRv2 value at T2. This could be due to the fact that the bees of FUNG group at T2 were not exposed anymore to the fungicide, and they were recovering from the fungicide pulse exposure, while bees of the INS treatment were in contact with the pesticide for a prolonged period of time. As with the INS treatment, the IBRv2 value of the MIX treatment was higher at T2 than at T1. The MIX IBRv2 value confirms the results of FUNG at T2, indicating recovery from the fungicide pulse. The obtained MIX value is probably due to the insecticide action only. These results confirm that biomarkers can be a useful tool in the framework of pesticide risk assessment as an early warning signal of pesticide side effects on bees in post-registration monitoring programs.

## Conclusions

Our study demonstrates that exposure to the commercial insecticide Confidor® and fungicide Folicur® may impact the solitary bee *O. bicornis* at different levels of biological organization: from enzymatic responses to feeding rate and survival. Our results showed that (i) chronic exposure to residual concentrations of imidacloprid affected feeding and survival of this solitary bee; (ii) an acute exposure to a fungicide, considered safe to use during bloom, had a temporary sub-lethal impact; (iii) contrary to our expectation, the pulse of fungicide did not exacerbate the effects of imidacloprid. As for the molecular tools, one of the four biomarkers tested, AChE, was inhibited by the fungicide and the insecticide, showing promise as an indicator of sub-lethal effects in *O. bicornis*. The IBRv2 index proved to be a powerful tool to describe the toxicological status of *O. bicornis*, highlighting a good ability of the bees to recover from the fungicide pulse, while a chronic exposure to INS caused increased sub-lethal effects. No effects of the binary mixture were observed. Overall, this study provides evidence for improving the current risk assessment procedures by including sub-lethal endpoints and other bee species in addition to *A. mellifera*.

## Supplementary Information

Below is the link to the electronic supplementary material.

Supplementary file1 (DOCX 40 KB)

## Data Availability

The datasets generated by the current study will be available upon request to the corresponding authors.
