# Supplementary material for: Biochemical responses, feeding and survival in the solitary bee Osmia bicornis following exposure to an insecticide and a fungicide alone and in combination
Source: Environ Sci Pollut Res Int. 2022 Nov 16;30(10):27636–49. doi: 10.1007/s11356-022-24061-x (PMC9995414; doi:10.1007/s11356-022-24061-x)
Supplement: Supplementary file 1 — Supplementary file1 (DOCX 40 KB) [file 11356_2022_24061_MOESM1_ESM.docx]

**Supplementary materials**

**Feeding, survival and biochemical responses after the exposure to an insecticide and a fungicide alone and in combination in *Osmia bicornis***

Cátia A.H. Martins^a,1^, Ilaria Caliani^b,1^, Antonella D’Agostino^c^, Agata Di Noi^d,*^, Silvia Casini^b^, Martina Parrilli^a^, Celeste Azpiazu^e,f^, Jordi Bosch^g^, Fabio Sgolastra^a^

^a^ Department of Agricultural and Food Sciences, Alma Mater Studiorum Università di Bologna, Viale Fanin 42, 40127, Bologna, Italy.

^b^Department of Physical, Earth and Environmental Sciences, University of Siena, Via Mattioli 4, 53100 Siena, Italy.

^c^Department of Management and Quantitative Studies, University of Naples Parthenope, Napoli, Italy.

^d^Department of Life Sciences, University of Siena, via Mattioli, 4, 53100 Siena, Italy.

^e^Institute of Evolutionary Biology (CSIC-Universitat Pompeu Fabra), Passeig Marítim de la Barceloneta 37, Barcelona 08003, Spain.

^f^Universidad Politécnica de Madrid, 28040 Madrid, Spain.

^g^CREAF, Universitat Autònoma de Barcelona, 08193 Bellaterra, Spain.

* Corresponding author.

E-mail address: agata.dinoi@student.unisi.it (A. Di Noi).

^1^ Contributed equally.

Table A.1. Daily syrup consumption (µl day^−1^) in *Osmia bicornis* females orally exposed to various pesticide treatments. CTRL: chronic exposure to regular syrup; FUNG: chronic exposure to regular syrup with a pulse of tebuconazole (185 mg L^-1^) on day 3; INS: chronic exposure to syrup with imidacloprid (5 µg L^-1^); MIX: chronic exposure to syrup with imidacloprid (5 µg L^-1^) + a pulse of tebuconazole (185 mg L^-1^) on day 3. Pre-pulse: first 2 days of exposure; Pulse: day 3; Post-pulse: after the 3^rd^ up to the date of 50% mortality within each treatment; TOTAL: Daily syrup consumption from the beginning of exposure until the 50% mortality within each treatment.

|  | | Pre-pulse | | Pulse (day 3) | | Post-pulse | | TOTAL | |
| --- | --- | --- | --- | --- | --- | --- | --- | --- | --- |
| Treatment | **N** | **Mean (SD)** | **Median** | **Mean (SD)** | **Median** | **Mean (SD)** | **Median** | **Mean (SD)** | **Median** |
| CTRL | 25 | 110 (30.6) | 114.4 | 118 (27.3) | 120 | 59.4 (21.1) | 55.2 | 69.2 (16.2) | 64.4 |
| FUNG | 26 | 77.3 (32.0) | 81.9 | 25.6 (18.8) | 20 | 45.4 (27.6) | 49.1 | 52.2 (22.4) | 53.0 |
| INS | 26 | 26.9 (9.7) | 24.7 | 14.4 (9.79) | 11.3 | 11.1 (4.9) | 11.1 | 15.1 (4.1) | 15.6 |
| MIX | 24 | 24.8 (8.3) | 24.4 | 35.9 (14.3) | 40 | 11 (5.15) | 10.2 | 16.7 (4.8) | 16.0 |

Table A.2. Results of Mann-Whitney U Test (One-sided tests*) when syrup consumption is compared between pre- pulse and post pulse- time.

| Treatment | p-value |
| --- | --- |
| CTRL | 0.0000 |
| FUNG | 0.0003 |
| INS | 0.0000 |
| MIX | 0.0000 |

*Note: Ho: median of pre-pulse -post-pulse assessment = 0 vs. Ha: median of pre-pulse -post-pulse assessment > 0

Table A.3. Results of the Kernel regression analysis (dependent variable is daily syrup consumption).

|  | **Average** | **SE** | **t** | **P-value** |
| --- | --- | --- | --- | --- |
| **Body size** | 6.1768 | 6.64318 | 0.930 | 0.354 |

Table A.4. Results of Fleming-Harrington test for equality of survivor functions (middle differences).

| Treatment | Events observed | Events expected | Sum of ranks |
| --- | --- | --- | --- |
| CTRL | 38 | 38.70 | -1.4370414 |
| FUNG | 31 | 41.03 | -1.9626036 |
| INS | 33 | 27.79 | 1.1195858 |
| MIX | 28 | 22.48 | 2.2800592 |
| Total | 130 | 130 | 0 |
| χ^2^(3) = 15.90; Pr > χ^2^ = 0.0012 | | | |

Table A.5. Results of Cox regression model.

|  | **Haz. Ratio** | **SE** | **z** | **P>\|z\|** | **[95% Conf. Interval]** |
| --- | --- | --- | --- | --- | --- |
| **Body size** | 2.16989 | 2.474617 | 0.68 | 0.497 | 0.2321125 - 20.28509 |

Table A.6. Activity of four biomarkers (AChE is expressed as μmol^-1^ g tissue^-1^ min. CaE, GST and ALP are expressed as nmol min^-1^ mg^-1^ protein) in *Osmia bicornis* females orally exposed to various pesticide treatments. CTRL: chronic exposure to regular syrup; FUNG: chronic exposure to regular syrup with a pulse of tebuconazole (185 mg L^-1^) on day 3; INS: chronic exposure to syrup with imidacloprid (5 µg L^-1^); MIX: chronic exposure to syrup with imidacloprid (5 µg L^-1^) + a pulse of tebuconazole (185 mg L^-1^) on day 3. Measurements were taken at T1 (day 4 of exposure; A) and T2 (day 6 of exposure; B).

|  |  | **AChE** | | | | | **CaE** | | | | | **GST** | | | | | **ALP** | | | | |
| --- | --- | --- | --- | --- | --- | --- | --- | --- | --- | --- | --- | --- | --- | --- | --- | --- | --- | --- | --- | --- | --- |
| **Time** | **Treatment** | **N** | **Min** | **Max** | **Mean (SD)** | **Median** | **N** | **Min** | **Max** | **Mean (SD)** | **Median** | **N** | **Min** | **Max** | **Mean (SD)** | **Median** | **N** | **Min** | **Max** | **Mean (SD)** | **Median** |
| **A** | **CTRL** | 8 | 1.45 | 2.86 | 2.09 (0.48) | 1.99 | 6 | 1.41 | 3.42 | 2.62 (0.73) | 2.74 | 5 | 602.21 | 3058.69 | 1568.73 (1021.93) | 1488.62 | 5 | 85.15 | 125.32 | 106.15 (15.94) | 110.59 |
|  | **FUNG** | 10 | 1.07 | 2.12 | 1.48 (0.35) | 1.44 | 6 | 1.31 | 4.61 | 2.41 (1.26) | 2.13 | 6 | 700.22 | 6344.31 | 2240.18 (2201.32) | 1144.76 | 6 | 14.58 | 66.74 | 42.72 (21.40) | 41.77 |
|  | **INS** | 10 | 0.83 | 2.03 | 1.34 (0.42) | 1.41 | 10 | 1.64 | 3.19 | 2.51 (0.48) | 2.54 | 5 | 600.50 | 1201.85 | 886.56 (270.03) | 800.14 | 2 | 21.71 | 109.15 | 65.43 (61.83) | 65.43 |
|  | **MIX** | 10 | 0.52 | 2.01 | 1.55 (0.48) | 1.75 | 9 | 0.61 | 5.73 | 2.88 (1.50) | 2.34 | 4 | 601.54 | 3119.65 | 1646.14 (1112.35) | 1431.69 | 3 | 16.80 | 144.29 | 69.15 (66.73) | 46.37 |
| **B** | **CTRL** | 10 | 1.17 | 2.64 | 1.94 (0.42) | 1.94 | 9 | 0.17 | 20.1 | 4.06 (6.13) | 2.66 | 7 | 405.02 | 5457.02 | 2865.88 (1924.03) | 2230.08 | 7 | 13.64 | 198.33 | 109.47 (65.6) | 102.21 |
|  | **FUNG** | 10 | 0.91 | 1.85 | 1.33 (0.33) | 1.30 | 8 | 0.21 | 24.59 | 5.91 (7.95) | 2.86 | 8 | 466.39 | 11688.10 | 2593.15 (3855.46) | 992.15 | 6 | 20.15 | 145.41 | 70.09 (56.77) | 44.48 |
|  | **INS** | 9 | 0.72 | 1.39 | 1 .00 (0.21) | 0.98 | 9 | 1.06 | 8.02 | 3.23 (2.28) | 2.27 | 5 | 521.50 | 1228.36 | 962.82 (297.54) | 1129.54 | 5 | 13.25 | 43.12 | 27.74 (13.21) | 26.43 |
|  | **MIX** | 4 | 0.23 | 2.13 | 1.23 (1.02) | 1.28 | 2 | 1.40 | 62.29 | 31.85 (43.06) | 31.85 | 5 | 939.77 | 9160.21 | 3679.79 (3295.66) | 3190.55 | 5 | 15.60 | 118.65 | 61.33 (38.37) | 52.05 |

Table A.7. Results of Kruskal-Wallis tests at T1 and T2 for the different tests: TSC (Mean daily syrup consumption; expressed as µl day−1); AChE (acetylcholinesterase; expressed as μmol^-1^ g tissue^-1^ min); CaE (carboxylesterase; expressed as nmol min^-1^ mg^-1^ protein); GST (glutathione-S-transferase; expressed as nmol min^-1^ mg^-1^ protein) and ALP (alkaline phosphatase; expressed as nmol min^-1^ mg^-1^ protein).

| Biomarkers | p-value at TIME 1 | p-values at TIME 2 |
| --- | --- | --- |
| TSC | 0.0001 | 0.0001 |
| AChE | 0.0194 | 0.0023 |
| CaE | 0.8263 | 0.8770 |
| GST | 0.5519 | 0.1852 |
| ALP | 0.1138 | 0.0885 |

Table A.8. Results of Dunn's Pairwise in comparison with the control group for TSC (Mean daily syrup consumption; expressed in µl day−1), AChE (acetylcholinesterase; expressed as μmol^-1^ g tissue^-1^ min), CaE (carboxylesterase; expressed as nmol min^-1^ mg^-1^ protein), GST (glutathione-S-transferase; expressed as nmol min^-1^ mg^-1^ protein) and ALP (alkaline phosphatase; expressed as nmol min^-1^ mg^-1^ protein).

| Biomarkers | TIME1 | | | TIME2 | | |
| --- | --- | --- | --- | --- | --- | --- |
|  | **FUNG** | **INS** | **MIX** | **FUNG** | **INS** | **MIX** |
| TSC | 0.2927 | 0.0001 | 0.0012 | 0.2580 | 0.0000 | 0.0002 |
| AChE | 0.0403 | 0.0074 | 0.0956 | 0.0607 | 0.0005 | 0.1129 |
| CaE | n.s. | n.s. | n.s. | n.s. | n.s. | n.s. |
| GST | n.s. | n.s. | n.s. | n.s. | n.s. | n.s. |
| ALP | n.s. | n.s. | n.s. | n.s. | n.s. | n.s. |
